# Extremely High *Tp53* Mutation Load in Esophageal Squamous Cell Carcinoma in Golestan Province, Iran

**DOI:** 10.1371/journal.pone.0029488

**Published:** 2011-12-27

**Authors:** Behnoush Abedi-Ardekani, Farin Kamangar, Masoud Sotoudeh, Stephanie Villar, Farhad Islami, Karim Aghcheli, Dariush Nasrollahzadeh, Noushin Taghavi, Sanford M. Dawsey, Christian C. Abnet, Stephen M. Hewitt, Saman Fahimi, Farrokh Saidi, Paul Brennan, Paolo Boffetta, Reza Malekzadeh, Pierre Hainaut

**Affiliations:** 1 Digestive Disease Research Center, Shariati Hospital, Tehran University of Medical Sciences, Tehran, Iran; 2 International Agency for Research on Cancer, Lyon, France; 3 Social Security Organization, Tehran, Iran; 4 Division of Cancer Epidemiology and Genetics, National Cancer Institute, Bethesda, Maryland, United States of America; 5 Department of Public Health Analysis, School of Community Health and Policy, Morgan State University, Baltimore, Maryland, United States of America; 6 Tisch Cancer Institute, Mount Sinai School of Medicine, New York, New York, United States of America; 7 Department of Medical Epidemiology and Biostatistics, Karolinska Institutet, Stockholm, Sweden; 8 Tissue Array Research Program, Laboratory of Pathology, Center for Cancer Research, National Cancer Institute, Bethesda, Maryland, United States of America; 9 Department of Public Health and Primary Care, University of Cambridge, Cambridge, United Kingdom; 10 International Prevention Research Institute, Lyon, France; Howard University, United States of America

## Abstract

**Background:**

Golestan Province in northeastern Iran has one of the highest incidences of esophageal squamous cell carcinoma (ESCC) in the world with rates over 50 per 100,000 person-years in both sexes. We have analyzed *TP53* mutation patterns in tumors from this high-risk geographic area in search of clues to the mutagenic processes involved in causing ESCC.

**Methodology/Principal Findings:**

Biopsies of 119 confirmed ESCC tumor tissue from subjects enrolled in a case-control study conducted in Golestan Province were analyzed by direct sequencing of *TP53* exons 2 through 11. Immunohistochemical staining for p53 was carried out using two monoclonal antibodies, DO7 and 1801. A total of 120 *TP53* mutations were detected in 107/119 cases (89.9%), including 11 patients with double or triple mutations. The mutation pattern was heterogeneous with infrequent mutations at common *TP53* “hotspots” but frequent transversions potentially attributable to environmental carcinogens forming bulky DNA adducts, including 40% at bases known as site of mutagenesis by polycyclic aromatic hydrocarbons (PAHs). Mutations showed different patterns according to the reported temperature of tea consumption, but no variation was observed in relation to ethnicity, tobacco or opium use, and alcoholic beverage consumption or urban versus rural residence.

**Conclusion/Significance:**

ESCC tumors in people from Golestan Province show the highest rate of *TP53* mutations ever reported in any cancer anywhere. The heterogeneous mutation pattern is highly suggestive of a causative role for multiple environmental carcinogens, including PAHs. The temperature and composition of tea may also influence mutagenesis.

## Introduction

Esophageal Cancer is common in Persia (current Iran) at least since the writings of the famous Iranian physician Avicenna (980–1037), who provided one of the earliest descriptions of this disease [1]. Since then, several authors have documented this pathology in a broad region extending from the eastern shores of the Caspian Sea to Central China, forming the “Central Asian Esophageal Cancer Belt” [2]. This region concentrates over half of the cases of esophageal squamous cell carcinoma (ESCC) detected in the world [2]. Other regions of high incidence have been identified in southern and eastern Africa and parts of South America [3]. In the high–incidence areas of northern Iran (Golestan) and central China, incidence rates of 50 to 100 per 100,000 person-years have been reported, as compared to rates varying between 2 and 30 per 100,000 person-years in Europe and the United States [4]. In Western countries, consumption of tobacco and alcohol, alone or in combination, are the main risk factors, and there is an excess of cases in males in agreement with the higher prevalence of these risk factors among men [3], [4]. In Golestan, however, tobacco and alcohol do not seem to play a prominent role [5], [6] and the best documented risk factors are the consumption of hot beverages [7], low socio-economic status [8] and, possibly, cultural or lifestyle habits such as the use of opium derivatives [5], [9]. However, these risk factors only partially explain the high incidence rates, and the main carcinogen(s) involved have not been identified. Given the late detection and poor cure rate of ESCC, the main hope for reducing mortality lies into prevention based on a better understanding of the specific causes of this cancer.

In several cancers, the tumor suppressor gene *TP53* (OMIM # 191170) shows patterns of mutations reflecting particular mutagenic exposures [10]. For example, in lung tumors from heavy smokers, a common pattern of transversions occurring at G∶C bases (G∶C to T∶A) is often detected [11]. These transversions are located at defined codons (mainly 157, 158, 245, 248 and 273) which have experimentally been demonstrated to be sites for DNA adduct formation by B[a]P metabolites [11]–[13]. In ESCC, studies of *TP53* mutation patterns have shown significant differences between cancers from low and high incidence areas. In 2001, two studies reported a description of *TP53* mutation patterns in retrospective series of ESCC from referral hospitals in Tehran, Iran, an area with medium risk of ESCC. Compared to ESCC from elsewhere, tumors from Tehran had significantly higher rate of G∶C to A∶T transitions at CpG dinucleotides, a mutation type commonly detected in cancers associated with inflammatory conditions [14], [15]. However, no systematic study has been performed to assess *TP53* mutation patterns in ESCC from Golestan Province, the center of the high risk region.

In this study, we used tumor tissues from ESCC cases with detailed annotations on pathology, individual history of exposure and ethnicity to assess *TP53* mutation patterns by sequencing and p53 protein expression by immunohistochemistry (IHC). We report the highest rate of somatic *TP53* mutations ever reported for any cancer, further supporting the notion that ESCC in this area develops as the consequence of heavy exposure to environmental mutagens, and providing molecular clues for potentially preventable risk factors.

## Results

### Patients

**Figure 1:**
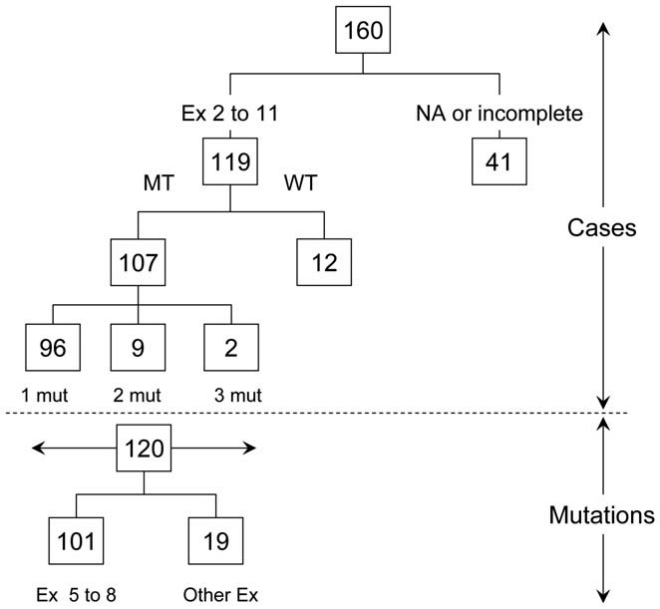
Flow chart summarizing the analyses carried out on ESCC cases used from the Golestan Case-Control Study and the number of mutations detected. Of a total of 160 cases, 119 were fully sequenced for exons (Ex) 2–11 whereas 41 were either not available (NA) or incompletely sequenced. Numbers of samples with mutations (MT) or with wild-type TP53 (WT) are indicated. Among MT cases, the number of cases with 1, 2 or 3 mutation(s) (mut) is given. Below the dotted line, the total number of mutations (120) is broken down into mutations in exons (Ex) 5 to 8 or in other exons.

Figure 1 summarizes the analyses conducted on ESCC cases from Golestan. Of the initial 160 biopsies, each from a different patient, DNA was successfully amplified for all *TP53* coding exons in 119. These patients were selected as subjects for this study and the rest (n = 41), for whom complete amplifications of all exons was not possible, were excluded. Table 1 shows the main characteristics of these 119 patients. The mean age and the sex distribution are in agreement with data previously reported from Golestan, where there is an approximately equal incidence of ESCC in males and females [16]. Of these patients, 65 (54.6%) were Turkmen and 54 (45.4%) were of non-Turkmen ethnicity, including Fars (26: 21.8%), Balooch (4: 3.4%), Turk (15: 12.6%), Sistani (7: 5.9%) and Kurd (2: 1.7%). The proportion of Turkmens among the cases was similar to the estimated proportion of ethnic Turkmens in the catchment area of the case-control study (55%; unpublished data). All cancers were invasive, with sizes ranging from 3 to 11 cm, including 89 patients (83.2%) diagnosed with stage III or IV tumors.

**Table 1:** Number of participants and mutations by demographic, lifestyle, and tumor characteristics.

Charactersitic^a^	Subcategory	All participantsNo (%^b^)	Participants with mutation No (%^c^)	Total No. of mutations^d^
**Mean age (SD); years**		65.5 (11.0)	65.3 (11.0)	-
**Age group**	<66 years	58 (48.7)	54 (93.1)	60
	≥66 years	61 (51.3)	53 (86.9)	60
**Sex**	Women	60 (50.4)	52 (88.1)	62
	Men	59 (49.6)	55 (91.7)	58
**Ethnicity**	Non-Turkmen	54 (45.4)	50 (92.6)	58
	Turkmen	65 (54.6)	57 (87.7)	62
**Place of residence**	Rural	85 (71.4)	77 (90.6)	88
	Urban	34 (28.6)	30 (88.2)	32
**Alcohol drinking**	Never	114 (95.8)	103 (90.4)	115
	Ever	5 (4.2)	4 (80.0)	5
**Tobacco/opium use**	Neither	67 (56.3)	61 (91.0)	69
	Tobacco	16 (13.5)	12 (75.0)	15
	Opium	15 (12.6)	15 (100)	16
	Both	21 (17.6)	19 (90.5)	20
**Tea temperature** e	≥4	46 (43.4)	44 (95.7)	47
	2–3	43 (40.6)	39 (90.7)	45
	0–1	17 (16.0)	13 (76.5)	15
**Tumor size**	<5 cm	27 (24.7)	25 (92.6)	28
	5–8 cm	67 (61.5)	62 (92.5)	70
	>8 cm	15 (13.8)	12 (80.0)	14
**Tumor stage**	I	4 (3.8)	3 (75.0)	3
	II	14 (13.0)	12 (85.7)	14
	III	37 (34.6)	32 (86.5)	38
	IV	52 (48.6)	49 (94.2)	55

a Because of missing values, the total number of participants for some variables is less than 119.

b Column percentages.

c Row percentages.

d The total number of mutations was 120, detected in 107 participants (see Figure 1).

e Estimated by the time interval between tea being poured and drunk (in minutes).

Among well-documented risk factors for ESCC, information on hot tea drinking was available for 106 subjects, among whom 43.4% reported drinking tea 4 minutes or more after pouring. Only 4.2% of patients reported ever using alcohol. The use of tobacco or opium products in various forms (smoking or chewing) was reported by 52 subjects (43.7%), among whom 21 (17.6%) reported using both substances. The majority of the subjects (71.4%) were from rural areas, with farming and/or raising animals as the main occupations.

### *TP53* mutation analysis

**Figure 2:**
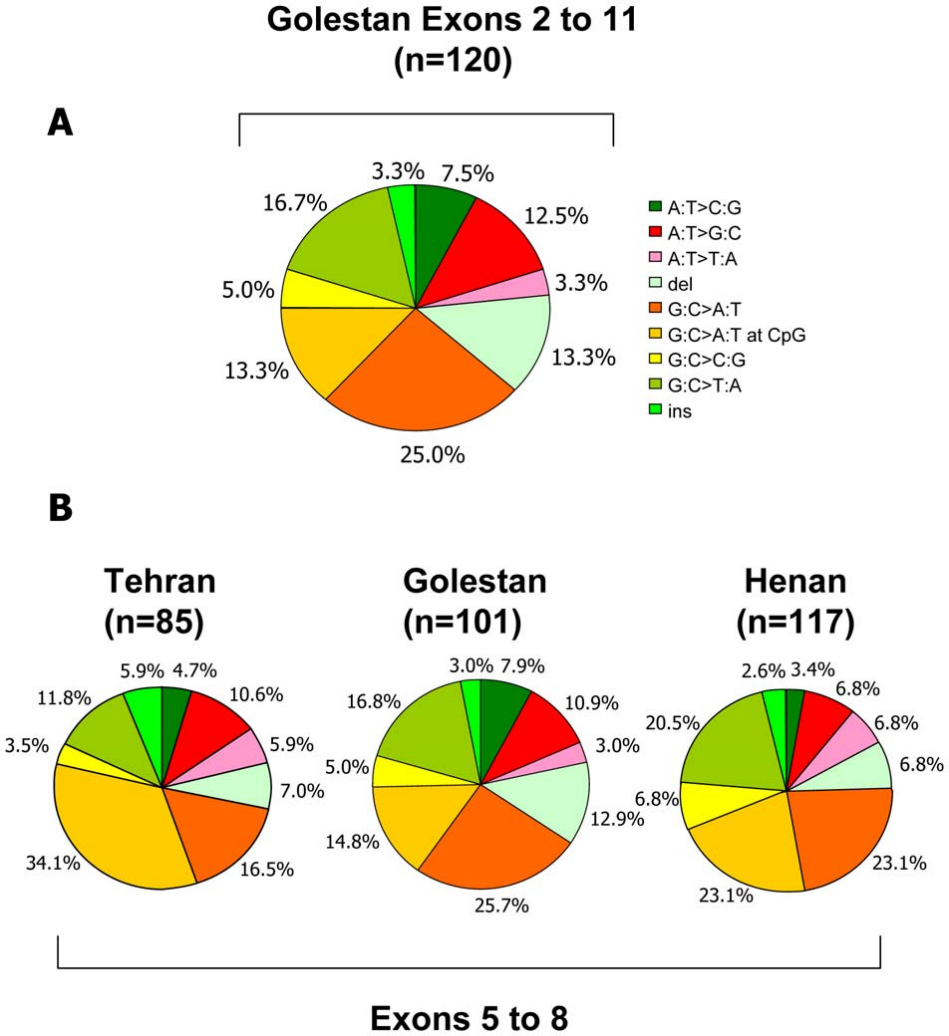
Distribution of *TP53* mutation types in ESCC. A: *TP53* Mutation types in ESCC from Golestan, exons 2 to 11. Mutations are grouped in categories as defined in the IARC *TP53* mutation database (http://www-p53.iarc.fr). B: Comparison of mutation types in exons 5 to 8 of ESCC from Golestan (this study, n = 101) with results from Tehran (n = 85) and Henan Province, China (n = 117) compiled in the IARC *TP53* mutation database (http://www-p53.iarc.fr/AdvancedCriteria.asp; search criteria: Esophagus, exons 5 to 8, Squamous Cell Carcinoma, surgery or biopsy, DNA; for China: Linxian and/or Henan, for Iran: Tehran).

Somatic *TP53* mutations were confirmed in 107 patients (89.9%) including nine patients with two different mutations and two patients with three mutations (total: 120 mutations; see detailed mutation description in Table S2). Figure 2A shows the distribution of the types of the 120 mutations in exons 2 through 11 (including splice junctions). The most common mutation type was G∶C to A∶T transitions (46; 38.3%), one third of them at CpG dinucleotides (16; 13.3%), followed by G∶C to T∶A transversions (20; 16.7%). Deletions represented 13.3% of all detected mutations whereas other mutation types (G∶C to C∶G, mutations at A∶T base pairs) were less frequent. There was no association between mutations and tumor grade or stage.

### Codon distribution

Like previous studies, the majority of the mutations (101 out of 120; 84.2%) were detected in exons 5–8 (Figure 1). These included mutations in exon 5 (29; 24.2%), exon 6 (25; 20.8%), exon 7 (25; 20.8%) and exon 8 (22; 18.3%). A total of 19 (15.8%) of the mutations were detected on exons 4, 9 and 10 while no mutation was found in exons 2 or 3. Only 9 (7.5%) of the mutations occurred at “hotspot” *TP53* codons (codons 175, 245, 273 and 282 where 20% of known *TP53* mutations occur in all cancers). No individual codon contained more than 4 mutations. Among the G∶C to T∶A mutations, 8/20 (40%) occurred at codons previously described as sites of PAH adduct formation which are commonly mutated in lung cancer of smokers.

### Comparing mutation patterns in Golestan and other areas

Table 2 compares the patterns of mutations of ESCC from Golestan with previously published mutation patterns from Tehran. Since the previous studies examined only exons 5–8 of *TP53*, for comparability, this part of analysis was done only on mutations in these exons (101 of the total 120 mutations, Figure 1). To compare data from Tehran and Golestan, mutations were categorized into six groups (Table 2), which showed a global *p* (with 5 degrees of freedom) of 0.06. A difference was found between the proportion of transition mutations at CpG dinucleotides in cases from the two areas (post-hoc analysis, *p* = 0.002; Table 2). Differences in the proportion of other mutations were not significant. Figure 2B further illustrates the pattern of mutations in exons 5–8 in Tehran, in Golestan, and in the Henan Province of China, another area of high incidence. Of these three patterns, the one of Henan showed the largest proportion of G∶C to T∶A and G∶C to C∶G transversions (20.5% and 7%, respectively) whereas the one of Tehran showed the lowest proportion of those mutations (12% and 3.5%), respectively. With 17% and 5%, respectively, the proportion of those transversions of Golestan was somewhat closer to Henan than to Tehran. Furthermore, the overall pattern of mutations in ESCC from Golestan was not statistically different from the one of ESCC from Henan.

**Table 2:** Comparison between *TP53* mutation patterns in ESCC of patients from Golestan and Tehran^a^.

Mutation Type	N° (%) Gonbad	N° (%) Tehran	p-value(b)
A∶T>C∶G	8 (7.9)	4 (4.7)	0.920(c)
A∶T>G∶C	11 (10.9)	9 (10.6)	
A∶T>T∶A	3 (3.0)	5 (5.9)	
Others	16 (15.8)	11 (12.9)	0.576
G∶C>A∶T	26 (25.7)	14 (16.5)	0.125
G∶C>A∶T at CpG	15 (14.8)	29 (34.1)	0.002
G∶C>C∶G	5 (5.0)	3 (3.5)	0.634
G∶C>T∶A	17 (16.8)	10 (11.8)	0.328

a : data for Tehran are from Sepehr et al., 2001 and Biramijamal et al. 2001 (see text).

b : p for the difference in prevalence of mutations in exons 5 through 8 in Golestan and Tehran, calculated by using the Pearson chi-square test.

c : *p* for all types of mutations at A∶T base pairs (often interpreted as caused by acetaldehyde, a metabolite of alcohol) taken as a single category.

### Association between mutations and risk factors

**Figure 3:**
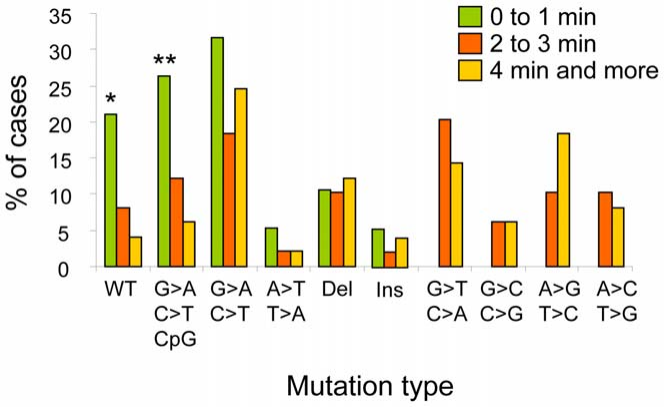
Distribution of *TP53* mutation types in ESCC from Golestan according to tea temperature. Tea temperature was estimated by the time interval between pouring and drinking tea (from 0–1 min to 4 min or more). Mutations are grouped in categories as in Figure 2. *: OR: 6.27, 95% CI, 1.04–37.69 for wild-type *TP53* in Group 0–1 min as compared to Group 4 min or more; **: OR: 6.40, 95% CI, 1.16–36.16 for G∶C to A∶T at CpG in Group 0–1 min as compared to Group 4 min or more. Note the absence of G∶C to T∶A, G∶C to C∶G, A∶T to G∶C and A∶T to C∶G mutations in Group 0–1 min.

Table 3 compares the three most abundant mutation types to the patient characteristics listed in Table 1. There was a small but significant inverse association between age and G∶C to T∶A transversions (using age as a continuous variable, adjusted OR: 0.93, 95% Cl, 0.88–0.99). Mutation types also showed differences according to the temperature of tea consumption, with G∶C to A∶T mutations at CpG sites being significantly more common among hot tea drinkers (within 0–1 min of pouring) than among subjects who drank tea at lower temperature (≥4 minutes after pouring; adjusted OR: 6.40, 95% CI, 1.16–35.16) Wild-type *TP53* was more common in hot tea drinkers (OR: 6.27, 95% CI, 1.04–37.69). In contrast, G∶C to T∶A, G∶C to C∶G, A∶T to G∶C and A∶T to C∶G mutations were found only in subjects who reported drinking tea ≥2 minutes after pouring (Figure 3). Overall, these observations suggest that there is an association between the temperature of tea consumption and the presence and types of *TP53* mutation.

**Table 3:** Associations between patient's characteristics and selected mutation types^a^.

	G∶C to A∶T			G∶C to A∶T at CpG			G∶C to T∶A		
Characteristic	No (% )	Crude OR (95% CI)	Adjusted OR (95% CI)	No (%^a^)	Crude OR (95%CI)	Adjusted OR (95%CI)	No (%^a^)	Crude OR (95%CI)	Adjusted OR (95%CI)
**Age (continuous)**	-	1.01 (0.97–1.05)	1.01 (0.97–1.06)	-	1.01 (0.96–1.06)	1.00 (0.95–1.06)	-	0.95 (0.91–1.00)	*0.93 (0.88–0.99)*
**Age group**									
<66 years	15 (23.4)	Ref	Ref	8 (12.5)	Ref	Ref	13 (20.3)	Ref	Ref
≥66 years	15 (22.1)	0.92 (0.41–2.09)	0.81 (0.33–1.99)	8 (11.8)	0.93 (0.33–2.66)	0.75 (0.23–2.49)	7 (10.3)	0.45 (0.17–1.21)	0.37 (0.11–1.25)
**Sex**									
Female	13 (20.6)	Ref	Ref	6 (9.5)	Ref	Ref	9 (14.3)	Ref	Ref
Male	17 (24.6)	1.26 (0.55–2.85)	1.31 (0.47–3.65)	10(14.5)	1.61 (0.55–4.72)	1.26 (0.32–5.00)	11 (15.9)	1.14 (0.44–2.96)	0.93 (0.23–3.72)
**Ethnicity**									
Non-Turkmen	14 (22.6)	Ref	Ref	8 (12.9)	Ref	Ref	10 (16.1)	Ref	Ref
Turkmen	16 (22.9)	1.02 (0.45–2.30)	0.72 (0.28–1.83)	8 (11.4)	0.87 (0.31–2.48)	0.52 (0.15–1.84)	10 (14.3)	0.87 (0.33–2.25)	1.33 (0.39–4.48)
**Place of residence**									
Rural	25 (26.0)	Ref	Ref	12 (12.4)	Ref	Ref	13 (13.4)	Ref	Ref
Urban	5 (13.9)	0.46 (0.16–1.31)	0.59 (0.16–2.12)	4 (11.4)	0.88 (0.26–2.91)	0.50 (0.08–3.06)	7 (20.0)	1.54 (0.56–4.24)	2.02 (0.48–8.45)
**Tobacco/opium use**									
Neither	19 (25.3)	Ref	Ref	8 (10.7)	Ref	Ref	14 (18.7)	Ref	Ref
Tobacco	3 (15.8)	0.55 (0.15–2.11)	0.20 (0.23–1.74)	1 (5.3)	0.47 (0.05–3.97)	0.80 (0.08–8.45)	1 (5.3)	0.24 (0.03–1.97)	Not converged
Opium	4 (25.0)	0.98 (0.28–3.41)	0.62 (0.15–2.49)	3 (18.7)	1.93 (0.45–8.27)	3.09 (0.60–15.90)	1 (6.2)	0.29 (0.04–2.39)	0.30 (0.34–2.72)
Both	4 (18.2)	0.65 (0.20–2.18)	0.56 (0.14–2.27)	4 (18.2)	1.86 (0.50–6.88)	2.97 (0.56–15.91)	4 (18.2)	0.97 (0.28–3.31)	0.82 (0.16–4.16)
**Hot tea consumption** b									
≥4	12 (24.5)	Ref	Ref	3 (6.1)	Ref	Ref	7 (14.3)	Ref	Ref
2–3	9 (18.4)	0.69 (0.34–1.84)	0.73 (0.26–2.08)	6 (12.2)	2.14 (0.50–9.09)	2.22 (0.49–10.17)	10 (20.4)	1.54 (0.53–4.44)	1.98 (0.57–6.86)
0–1	6 (31.6)	1.42 (0.44–4.57)	1.12 (0.32–3.94)	5 (26.3)	*5.48 (1.16–25.84)*	*6.40 (1.16–35.16)*	0 (0.0)	(-)	(-)

a Three selected types of mutations (see text) were compared to other mutations and wild-types. ORs and 95% CIs were calculated using logistic regression models. In the adjusted models, results were adjusted for other variables in the table except for age group (categorical); results for age group were not adjusted for the age (continuous) variable.

b Interval between tea being poured and drunk (in minutes). Data were available for 106 patients.

### p53 protein expression

The expression of p53 protein was evaluated by IHC on ethanol-fixed tumor tissue microarrays, using two antibodies, DO7 and 1801. Cores of 12 cases were lost during sectioning and/or staining, so IHC was evaluable for only 107/119 cases. A good agreement was found between the results of the two antibodies (88.8% agreement with weighted k = 0.73). Of the 50 evaluable cases with missense mutation, 41 (82%) were positive for p53 (strong or moderate staining) and 9 were negative (negative or weak staining). In contrast, among cases with mutations predicting absence of p53 protein (nonsense, splice, or frameshift mutations), 28 (77.8%) of 36 evaluable cases were negative (no or weak staining), whereas only 8 (22.2%) were positive (strong or moderate staining) (Table S3). Thus, overall, there was a highly significant association between the presence of missense mutation and p53 expression scores (*p*<0.001), and there was a strong association between nonsense/splice/frameshift mutations and lack of p53 expression (*p* = 0.013/*p* = 0.005/*p* = 0.018 respectively).

## Discussion

ESCC is by far the most common cancer in both males and females in Northern Iran as well as in large parts of Central Asia [9]. The main challenge to reduce the incidence and the mortality of this extremely lethal disease is to develop prevention strategies based on a precise understanding of environmental causes. As an approach to identify new clues for the mechanisms of mutagenesis leading to ESCC in that part of the world, we have analyzed for the first time the patterns of mutations in the tumor suppressor gene *TP53*. All mutations were identified by direct sequencing and were confirmed by at least one second independent sequencing of a distinct PCR product. In this study, fixation procedures and timing were also carefully controlled. Moreover, statistical analysis did not show any association between mutation type and type of fixative, even after adjustment for age, sex, ethnicity, place of residence, tobacco/opium use and tea consumption time (data not shown). Thus, the mutations reported here are unlikely to be biased by sequencing or tissue fixation artifacts.

We detected at least one mutation in 89.9% of the cases (107/119), a prevalence which is, to our knowledge, the highest ever reported in any cancer anywhere. For comparison, cancers with particularly high mutation rates include lung cancers of very heavy smokers (80%; reviewed in reference [11]) or liver cancers in subjects heavily exposed to aflatoxin (60–75%; reviewed in reference [17]). In ESCC from other areas of the world, prevalence varying between 35% (Lyon, Southeastern France) [18] and 80% (Normandy, Northwestern France) [19] have been reported. Mutation prevalence appears to be consistently high in areas of high incidence, as for example parts of China [20] or Southern Brazil [21], [22]. Two previous studies on ESCC cases recruited in Tehran showed a mutation prevalence of 50%, [14], [15] substantially lower than the prevalence we found in our cases from Golestan. Moreover, in Golestan, 10% of patients with mutations showed more than one mutation in *TP53*.

The *TP53* mutation pattern in ESCC from Golestan is heterogeneous, with fewer than 10% of the mutations occurring at *TP53* “hotspot” codons (for reference mutation patterns see IARC *TP53* database at http://www.iarc.fr/p53). The most common mutation type was G∶C to A∶T transitions at positions that do not contain CpGs, a type of mutations which can be induced by multiple mechanisms and is therefore difficult to assign to any specific risk factor. *In vitro*, there is experimental evidence that guanines in such positions are targeted for adduct formation by nitrosamines such as 4-(methylnitrosamino)-1-(3-pyridyl)-1-butanone (NNK) [23]. Interestingly, a high proportion of G∶C to A∶T transitions has also been observed in ESCC in other areas of high incidence [20]–[22]. Overall, mutations attributable to endogenous mutagenic processes were infrequent; for example, G∶C to A∶T mutations at CpG sites, a type of mutation which often occurs as the result of spontaneous deamination of 5′methylcytosine, represented only 13.3% of all mutations, in contrast to 25% of mutations in all cancers compiled in the *TP53* mutation database (n = 26597) and 33% of the mutations in ESCC cases from Tehran (n = 85) [14], [15]. On the other hand, transversion mutations, which often occur as the result of mutagenesis by carcinogens forming bulky DNA adducts, represented about 30% of all mutations. Among those, the most common type was G∶C to T∶A. This type of mutation is frequent in tumor types where PAHs play an important role, such as lung cancer in smokers [11]. In the present series, although G∶C to T∶A transversions are less frequent than in lung cancers of smokers [13], 40% of them occurred at codons described as sites of adduction of PAH. Interestingly, mutations at these sites were not observed in ESCC cases from Tehran [15]. Several lines of evidence have suggested a role for polycyclic aromatic hydrocarbons (PAHs) in the etiology of ESCC in Golestan. Dietary intake of benzo[a]pyrene (B[a]P) has been reported to be higher than in lower-risk Fars Province [24]. High levels of 1-hydroxypyrene glucuronide (1-OHPG), a biomarker of PAH exposure, were detected in urine samples in 83% of participants from Golestan [25]. In a recent study, we used antibodies to PAH-adducts to assess exposure in biopsies of histopathologically normal esophagus from ESCC cases and controls from Golestan. We found a strong and significant association between the intensity of staining and case status (adjusted OR for the 5^th^ quintile: 26.6 (95% CI, 5.21 to 135); *p* for trend <0.001) [26]. Our mutation data are consistent with a role for PAH in mutagenesis in ESCC in Golestan. However, the heterogeneity of the mutation pattern suggests that PAHs are only one of several types of mutagens and that other still-unidentified factors may play additional or independent roles.

PAHs are common agents produced during incomplete combustion of organic materials. Their major sources other than tobacco smoke are food products [27], air pollution [28] and occupational [29] exposures. IARC has classified B[a]P and several PAH mixtures as group 1 carcinogen for humans [30]. Further studies are needed to identify the source(s) of PAH associated with risk of ESCC in the population of Golestan.

Detailed analysis of the mutation pattern did not support the association of mutations with sex, tobacco or opium use, alcohol consumption, ethnicity, or urban vs. rural residence. A small but significant reduction in the rate of G∶C to T∶A transversions was observed with increasing age, suggesting that this type of mutation might be more common in cancers that develop at an early age. However, the biological basis of this association is not known.

The absence of association with opium deserves comment, since consumption of opium pyrolysates has been shown to be a risk factor in Golestan, and these pyrolysates contain a number of carcinogens including PAHs [31], [32]. In the current study, 36 patients reported using opium, including 21 who also reported tobacco use. Of the 15 patients who used only opium, all had a mutation in *TP53*, although the types of mutations were not significantly different than in patients who did not report opium use. This low number of patients reporting opium use is unlikely to be due to a reporting bias, since a previous study in a cohort setting in Golestan has confirmed the validity of this self-reported information using urinary metabolites as a control [33]. It should be noted that there is no evidence that opium usage leads to the formation of a particular type of mutation. Furthermore, the small number of opium users in this study may not be sufficient to detect an effect on mutation type, if any exists.

Our study revealed different mutation patterns related to tea drinking habits. Wild-type *TP53* and G∶C to A∶T transitions (in particular at CpG sites) were most often seen in subjects who reported drinking tea within 1 minute of pouring. Conversely, transversion mutations (including G∶C to T∶A mutations) were observed only in subjects who reported a preference for drinking tea with a longer delay (at least 2 minutes after pouring). A previous study has shown that this method of measuring time-to-drinking intervals was a reliable surrogate for actual tea temperature. Using this method, Islami *et al.*, (2009) have shown that tea temperature was a significant risk factor for ESCC in Golestan [7]. Our results further suggest that tea temperature affects mutagenic processes in this population. Surprisingly patients with wild-type *TP53* were exclusively found among drinkers of hot tea, suggesting that thermal injury may represent a very potent carcinogenic mechanism that do not require *TP53* mutation. On the other hand, time after pouring may affect the tea concentration in potential mutagens derived from tea leaves, as well as the release of other toxic agents (e.g. metal ions) from tea containers. Hot temperature may also affect tissue permeability, tissue damage and healing of the esophageal epithelium, thus modulating the way in which the epithelium acts as a barrier to carcinogens from tea or other sources [34].

A comparison between mutation patterns in Golestan and other areas of the world is only possible for mutations in exons 5–8, which are the only exons that have been analyzed in the majority of previously published studies (see Figure 2B). This comparison shows that the mutations in ESCC from Golestan are different from the ESCC mutations seen in Tehran, strengthening the hypothesis of specific risk factors in Golestan. The differences were less marked between the patterns of mutations in ESCC from Golestan and from Henan Province, China, another high incidence area. Interestingly, there are similarities between the epidemiology of ESCC in Golestan and in central China. In particular, high levels of PAHs have been identified in food from Linxian, Henan Province, and these carcinogens have also been proposed to contribute to that region's high incidence of esophageal cancer [35].

In conclusion, this study shows an extremely high prevalence of diverse types of *TP53* mutations in ESCC tumors from Golestan. Most of these mutations are missense, leading to the accumulation of mutant p53 protein. Thus, ESCC from Golestan might be responsive to treatment by drugs that rescue p53 protein function, such as PRIMA-^MET^, a drug that has been shown to induce massive apoptosis in cells that express mutant p53 proteins [36]. The complex mutation pattern cannot be ascribed to a single category of mutagens. A plausible model for ESCC carcinogenesis in this area may be that the population at risk is exposed to multiple factors that damage the esophageal mucosa (e.g. thermal stress, toxic agents or irritants). In such conditions, tissue healing and the DNA repair capacity of epithelial cells may be diminished, making them susceptible to mutagenesis caused by different types of carcinogens, even if present at only moderate levels. In the case of the most severe forms of thermal stress (the drinkers of hot tea), the contribution of such carcinogens may even be limited (as suggested by the high proportion of patients with wild-type *TP53* or with CpG mutations). Further studies are needed to analyze in detail the mechanisms causing the extremely high load of *TP53* mutation observed in ESCC cases from Golestan and to assess whether similar mechanisms may characterize ESCC carcinogenesis throughout the so-called “Central Asian Esophageal Cancer Belt”, an area encompassing several populations and extending from the Caspian Sea to the Sea of China in which ESCC is the most frequent type of cancer.

## Materials and Methods

### Ethics statement

Written consent was obtained from all participants in the Golestan Case-Control Study (GCCS) and this study was approved by the Institutional Review Boards of the United States National Cancer Institute (US NCI), IARC and the Digestive Disease Research Center (DDRC) of Tehran University of Medical Sciences (TUMS).

### Cases and tissue selection

ESCC cases came from the Golestan Case-Control Study (GCCS), a study conducted between 2003 and 2007. GCCS is part of the GEMINI project (Gastro-Eesophageal Malignancies In Northern Iran), a group of collaborative studies between US NCI, IARC and DDRC of TUMS. The study design, objectives and methods of the GCCS have been published previously [5], [9], [37]. The catchment area of the GCCS covered the urban and rural areas of Gonbad, Kalaleh, Minoodasht districts in the eastern part of Golestan. The population of these areas is about 50% ethnic Turkmens, a group of several tribes of central Asian ancestry in whom ESCC has been reported to be very frequent. The other 50% of the population includes different ethnic groups, mainly Fars, Turks and Baloochs. During interviews conducted in the case-control study, individual information was collected on demographics, nutrition, occupation, diet and lifestyle habits. In the present study, we included the following variables: age, sex, ethnicity (Turkmen/non-Turkmen); tobacco consumption (ever/never), opium consumption (ever/never), alcohol consumption (ever/never), tea temperature (estimated by the time interval between tea being poured and drunk, and categorized in minutes) and residence (urban/rural). Data on daily amount of tea consumption were not collected. Of note, the method for scoring tea temperature has been validated in a previous study which reported a strong agreement between responses to the questions on temperature at which tea was drunk and interval from tea being poured to being drunk (weighted kappa 0.68). [7] The GCCS recruited a total of 300 histologically confirmed ESCC cases. One hundred and sixty cases were chosen for this study based on available biopsies. To be included, a case must have had at least one large endoscopic tumor biopsy with at least 70% tumor cells. Biopsies were obtained in the course of chromoendoscopy using Lugol's iodine, fixed in 10% buffered formalin or 70% ethanol and embedded in paraffin. Diagnosis has performed independently by two pathologists on heamatoxylin-eosin (H&E) stained slides.

### Analysis of *TP53* mutations

Biopsies containing at least 70% of tumor cells (as determined by examination of H&E sections) were deparaffinized and DNA was extracted using QIAamp DNA MicroKit from QIAGEN (Hilden, Germany) as described by the manufacturer. *TP53* mutation analysis of exons 2 through 11 was performed by direct sequencing of PCR products encompassing the entire coding sequence and splice junctions. Sequencing was performed on an Applied Biosystems PRISM 3100 Genetic Analyzer (Applied Biosystems, Foster City, CA). Each PCR product was generated in duplicate, with one product sequenced in the forward direction and the other in the reverse direction. In the case of discordant sequencing results, the complementary sequences were analyzed and the final result was confirmed by sequencing a third, independent PCR product. Primers, PCR and sequencing conditions have been described in detail and are available on the *TP53* IARC database website at http://www-p53.iarc.fr/Download/TP53_DirectSequencing_IARC.pdf. Chromatograms were analyzed semi-automatically by visual inspection of sequences imported in sequence analysis software Seqscape (Applied Biosystems) using the reference sequence NC_000017.9 from Genbank (http://www-p53.iarc.fr/TP53sequence_NC_000017-9.html). Variations were checked with the mutation validation tool available at http://www-p53.iarc.fr/MutationValidationCriteria.asp. For comparison of results with sequencing data from ESCC from other geographic areas, the IARC *TP53* mutation database (version R15, November 2010, http://www.iarc.fr/p53) was used (Table S1).

### Immunohistochemistry

p53 protein expression was evaluated by IHC using two monoclonal antibodies, DO7 and 1801 which recognize distinct epitopes in the N-terminal domain of p53. Tumor tissue microarrays were prepared as previously described [26]. After deparaffinization and rehydration, antigen retrieval was carried out for 20 minutes using Trilogy solution (Cell Marque) in a steamer (95°C). Endogenous peroxidases were blocked with 3% H_2_O_2_ in methanol for 5 minutes. Sections were processed in an automated Dako stainer, with the following steps: incubation with primary antibodies (DO7, Dako; 1∶50, 30 minutes or 1801, Oncogene Science; 1∶40, 1 hour) at room temperature; incubation with the secondary antibody (Goat anti-Mouse, peroxidase-coupled, Dako, 1∶250) for 15 min at room temperature and visualization using 3, 3′-diaminobenzidine solution (DAB) for 15 minutes. Expression was scored using a composite score in which the percentage of stained tumor cells was evaluated on a scale from 0 to 4 (0: No staining; 1: 1–25%; 2: 26–50%, 3: 51–75%; 4: 76–100%) and the intensity of staining was assessed on a scale from 0 (Negative) to 3 (strong). The two scores were multiplied to generate a composite score (0–12) categorized in 4 groups: 0 = negative; 1–4 = weak; 5–8 = moderate; and 9–12 = strong.

### Statistical analysis

Stata Version 11 (StataCorp LP, College Station, TX) was used to conduct all statistical analyses. Two-sided *P*<0.05 was considered as statistically significant. Age was included in our analyses separately as continuous and categorical variables (greater than or equal to versus less than the median age (66 years)). Consistent with our previous studies, tea temperature was estimated by the time interval between tea being poured and drunk, and was categorized as 0–1, 2–3, and 4 minutes or more [7]. Tobacco and opium use variables were combined and categorized as never-use of tobacco and opium, ever-use of tobacco only, ever-use of opium only, and ever-use of both tobacco and opium [5]. The distribution of mutation types in Golestan was compared with those in Tehran and a high incidence area of China (Henan Province) using chi-square tests (data selected from the IARC *TP53* mutation database; http://www-p53.iarc.fr ). Odds ratios (OR) and 95% confidence intervals (CI) were calculated for the association between several patient characteristics and risk of selected mutations using multivariate logistic regression models, in which the following variables were included: age (continuous), sex, ethnicity (Turkmen vs. non-Turkmen), place of residence (urban vs. rural), tobacco and/or opium use, and tea temperature (time interval between tea being poured and drunk). For IHC, the agreement between results from the two anti-p53 antibodies was calculated with a weighted kappa statistic. The association between the presence of mutations and the results of IHC was examined using chi-square tests.

## Supporting Information

Table S1 **Source of data on** ***TP53*** **mutations in Henan Province (China) and in Tehran: List of articles and data compiled in the IARC** ***TP53*** **database.**
(DOC)

Table S2 **Detailed** ***TP53*** **Mutations description in ESCC cases.**
(DOC)

Table S3 **Correspondence between** ***TP53*** **mutation status and Immunohistochemical detection of p53 protein in ESCC cases.**
(DOC)
