# Supplementary material for: Extremely High Tp53 Mutation Load in Esophageal Squamous Cell Carcinoma in Golestan Province, Iran
Source: PLoS One. 2011 Dec 27;6(12):e29488. doi: 10.1371/journal.pone.0029488 (PMC3246475; doi:10.1371/journal.pone.0029488)
Supplement: Table S1 — Source of data on TP53 mutations in Henan Province (China) and in Tehran: List of articles and data compiled in the IARC TP53 database. (DOC) [file pone.0029488.s001.doc]

**Table S1: Source of data on *TP53* mutations in Henan Province (China) and in Tehran: List of articles and data compiled in the IARC *TP53* database**

| **First author** | **Publication year** | **PMID** | **No. of reported mutations** |
| --- | --- | --- | --- |
| *References for studies from Henan Province, China* | | | |
| Shi ST | 1996 | 0010223186 | 37 |
| Cao W | 2004 | 15305417 | 18 |
| Smeds J | 2002 | 11960918 | 14 |
| Gao H | 1994 | 0008044781 | 13 |
| Unpublished^a^ | - | - | 13 |
| Shi ST | 1996 | 0008895479 | 8 |
| Yuanyuan L | 1995 | - | 6 |
| Liang YY | 1995 | 0007768632 | 4 |
| Cai YC | 2000 | 0010753204 | 4 |
| Total No. of mutations | | | 117 |
| *References for studies from Iran, Tehran* | | | |
| Sepehr A | 2001 | 11704866 | 57 |
| Biramijamal F | 2001 | 11306496 | 28 |
| Total No. of mutations | | | 85 |

^a^: Hainaut and Martel, data generated at IARC using specimens from Linxian, Henan Province
