# Supplementary material for: Extremely High Tp53 Mutation Load in Esophageal Squamous Cell Carcinoma in Golestan Province, Iran
Source: PLoS One. 2011 Dec 27;6(12):e29488. doi: 10.1371/journal.pone.0029488 (PMC3246475; doi:10.1371/journal.pone.0029488)
Supplement: Table S2 — Detailed TP53 Mutations description in ESCC cases. (DOC) [file pone.0029488.s002.doc]

**Table S2: *TP53* Mutation description ^(a)^**

| Patient Number | Exon/Intron | g_description | c_description | p_description | Effect | Type |
| --- | --- | --- | --- | --- | --- | --- |
| 1 | 8-exon | g.13839G>A | c.859G>A | p.E287K | missense | G:C>A:T |
| 2 | 4-exon | g.11392_11398del7 | c.161del7 | p.? ^(b)^ | frameshift | deletion |
| 3 | 5-exon | g.12449G>T | c.461G>T | p.G154V | missense | G:C>T:A |
| 4 | 5-exon | g.12444G>C | c.456G>C | p.P152P | silent | G:C>C:G |
| 5 | 4-exon | g.11460C>A | c.229C>A | p.P77T | missense | G:C>T:A |
|  | 4-exon | g.11462A>C | 231A>C | p.P77P | silent | A:T>C:G |
| 6 | 6-exon | g.12682T>C | c.613T>C | p.Y205H | missense | A:T>G:C |
| 7 | i7-intron | g.13420G>A | c.782+1G>A | p.? | splice | G:C>A:T |
| 8 | 6-exon | g.12686del1 | c.617del1 | p.? | frameshift | deletion |
| 9 | 5-exon | g.12449G>T | c.461G>T | p.G154V | missense | G:C>T:A |
| 10 | 5-exon | g.12521A>C | c.533A>C | p.H178P | missense | A:T>C:G |
| 11 | 8-exon | g.13797C>A | c.817C>A | p.R273S | missense | G:C>T:A |
| 12 | 7-exon | g.13384G>T | c.747G>T | p.R249S | missense | G:C>T:A |
| 13 | 8-exon | g.13811T>C | c.831T>C | p.C277C | silent | A:T>G:C |
| 14 | 6-exon | g.12714T>G | c.645T>G | p.S215R | missense | A:T>C:G |
| 15 | 5-exon | g.12456C>T | c.468C>T | p.R156R | silent | G:C>A:T at CpG |
| 16 | 8-exon | g.13790del1 | c.810del1 | p.? | frameshift | deletion |
| 17 | 8-exon | g.13824C>T | c.844C>T | p.R282W | missense | G:C>A:T at CpG |
| 18 | i9-intron | g.14077T>C | c.993+12T>C | p.? | intronic | A:T>G:C |
| 19 | 7-exon | g.13379C>T | c.742C>T | p.R248W | missense | G:C>A:T at CpG |
| 20 | i7-intron | g.13420G>A | c.782+1G>A | p.? | splice | G:C>A:T |
| 21 | i7-intron | g.13420G>A | c.782+1G>A | p.? | splice | G:C>A:T |
| 22 | 6-exon | g.12741G>A | c.672G>A | p.E224E | silent | G:C>A:T |
| 23 | 7-exon | g.13399_13400ins1 | c.762_763ins1 | p.? | frameshift | insertion |
| 24 | 7-exon | g.13338A>G | c.701A>G | p.Y234C | missense | A:T>G:C |
|  | 7-exon | g.13342C>T | c.705C>T | p.N235N | silent | G:C>A:T |
| 25 | 5-exon | g.12393C>G | c.405C>G | p.C135W | missense | G:C>C:G |
| 26 | 6-exon | g.12659T>G | c.590T>G | p.V197G | missense | A:T>C:G |
| 27 | 7-exon | g.13380G>A | c.743G>A | p.R248Q | missense | G:C>A:T at CpG |
| 28 | 5-exon | g.12485C>A | c.497C>A | p.S166X | nonsense | G:C>T:A |
| 29 | 7-exon | g.13379C>T | c.742C>T | p.R248W | missense | G:C>A:T at CpG |
| 30 | 5-exon | g.12512G>A | c.524G>A | p.R175H | missense | G:C>A:T at CpG |
| 31 | 6-exon | g.12718del1 | c.649del1 | p.? | frameshift | deletion |
| 32 | 9-exon | g.14026del1 | c.954del1 | p.? | frameshift | deletion |
| 33 | 5-exon | g.12439C>A | c.451C>A | p.P151T | missense | G:C>T:A |
| 34 | 6-exon | g.12706C>T | c.637C>T | p.R213W | missense | G:C>A:T at CpG |
| 35 | 8-exon | g.13839G>T | c.859G>T | p.E287X | nonsense | G:C>T:A |
| 36 | 5-exon | g.12451A>C | c.463A>C | p.T155P | missense | A:T>C:G |
|  | 8-exon | g.13795T>G | c.815T>G | p.V272G | missense | A:T>C:G |
| 37 | i9-intron | g.14066G>C | c.993+1G>C | p.? | splice | G:C>C:G |
| 38 | 7-exon | g.13377_13382del6 | c.740_745del6 | p.? | in-frame | deletion |
| 39 | 4-exon | g.11504G>A | c.273G>A | p.W91X | nonsense | G:C>A:T |
| 40 | 5-exon | g.12505G>A | c.517G>A | p.V173M | missense | G:C>A:T |
|  | 6-exon | g.12727T>A | c.658T>A | p.Y220N | missense | A:T>T:A |
| 41 | 5-exon | g.12454C>G | c.466C>G | p.R156G | missense | G:C>C:G |
| 42 | 5-exon | g.12512G>A | c.524G>A | p.R175H | missense | G:C>A:T at CpG |
| 43 | 5-exon | g.12444G>C | c.456G>C | p.P152P | silent | G:C>C:G |
| 44 | i8-intron | g.13931G>A | c.919+32G>A | p? | intronic | G:C>A:T |
| 45 | 8-exon | g.13777G>A | c.797G>A | p.G266E | missense | G:C>A:T |
| 46 | 8-exon | g.13896C>T | c.916C>T | p.R306X | nonsense | G:C>A:T at CpG |
| 47 | 6-exon | g.12684T>G | c.615T>G | p.Y205X | nonsense | A:T>C:G |
| 48 | 8-exon | g.13836G>A | c.856G>A | p.E286K | missense | G:C>A:T |
| 49 | i9-intron | g.14067T>C | c.993+2T>C | p.? | splice | A:T>G:C |
| 50 | 6-exon | g.12682T>C | c.613T>C | p.Y205H | missense | A:T>G:C |
| 51 | 7-exon | g.13348G>A | c.711G>A | p.M237I | missense | G:C>A:T |
| 52 | 6-exon | g.12652A>T | c.583A>T | p.I195F | missense | A:T>T:A |
| 53 | 5-exon | g.12505G>T | c.517G>T | p.V173L | missense | G:C>T:A |
| 54 | 6-exon | g.12701C>T | c.632C>T | p.T211I | missense | G:C>A:T |
| 55 | 8-exon | g.13839G>T | c.859G>T | p.E287X | nonsense | G:C>T:A |
| 56 | 6-exon | g.12664del1 | c.595del1 | p.? | frameshift | deletion |
| 57 | 6-exon | g.12646_12648 | c.577_579del3 | p.? | in-frame | deletion |
|  | 6-exon | g.12718del1 | c.649del1 | p.? | frameshift | deletion |
| 58 | 8-exon | g.13821G>A | c.841G>A | p.D281N | missense | G:C>A:T |
| 59 | 4-exon | g.11476del1 | c.245del1 | p.? | frameshift | deletion |
| 60 | 5-exon | g.12391T>G | c.403T>G | p.C135G | missense | A:T>C:G |
| 61 | 5-exon | g.12457G>T | c.469G>T | p.V157F | missense | G:C>T:A |
| 62 | 7-exon | g.13344A>G | c.707A>G | p.Y236C | missense | A:T>G:C |
| 63 | 7-exon | g.13344A>G | c.707A>G | p.Y236C | missense | A:T>G:C |
| 64 | 6-exon | g.12655del1 | c.586del1 | p.? | frameshift | deletion |
| 65 | 7-exon | g.13409G>A | c.772G>A | p.E258K | missense | G:C>A:T |
| 66 | 5-exon | g.12526G>T | c.538G>T | p.E180X | nonsense | G:C>T:A |
| 67 | 5-exon | g.12505G>A | c.517G>A | p.V173M | missense | G:C>A:T |
| 68 | 5-exon | g.12538G>A | c.550G>A | p.D184N | missense | G:C>A:T |
| 69 | 7-exon | g.13382A>T | c.745A>T | p.R249W | missense | A:T>T:A |
| 70 | 5-exon | g.12505G>A | c.517G>A | p.V173M | missense | G:C>A:T |
| 71 | 7-exon | g.13362G>T | c.725G>T | p.C242F | missense | G:C>T:A |
| 72 | 4-exon | g.11560G>T | c.329G>T | p.R110L | missense | G:C>T:A |
| 73 | 5-exon | g.12390_12391ins2 | c.402_403ins2 | p.? | frameshift | insertion |
|  | 6-exon | g.12655C>T | c.586C>T | p.R196X | nonsense | G:C>A:T at CpG |
| 74 | 7-exon | g.13373del1 | c.736del1 | p.? | frameshift | deletion |
| 75 | 5-exon | g.12547del3 | c.559del3 | p.? | splice | deletion |
| 76 | 5-exon | g.12405_12413del9 | c.417_425del9 | p.? | in-frame | deletion |
| 77 | i4-intron | g.11608T>C | c.375+2T>C | p.? | splice | A:T>G:C |
| 78 | i4-intron | g.11608T>A | c.375+2T>A | p.? | splice | A:T>T:A |
| 79 | 6-exon | g.12655C>T | c.586C>T | p.R196X | nonsense | G:C>A:T at CpG |
| 80 | i6-intron | g.12742G>T | c.672+1G>T | p.? | splice | G:C>T:A |
| 81 | i8-intron | g.13991G>T | c.920-1G>T | p.? | splice | G:C>T:A |
|  | i9-intron | g.14066G>A | c.993+1G>A | p.? | splice | G:C>A:T |
|  | 9-exon | g.13992C>T | c.920C>T | p.A307V | missense | G:C>A:T |
| 82 | 8-exon | g.13896C>T | c.916C>T | p.R306X | nonsense | G:C>A:T at CpG |
| 83 | 6-exon | g.12728A>G | c.659A>G | p.Y220C | missense | A:T>G:C |
| 84 | 5-exon | g.12443C>G | c.455C>G | p.P152R | missense | G:C>C:G |
| 85 | i10-intron | g.17889C>T | c.1101-21C>T | p.? | intronic | G:C>A:T |
|  | 10-exon | g.16932ins1 | c.1041ins1 | p.? | frameshift | insertion |
| 86 | 8-exon | g.13794G>T | c.814G>T | p.V272L | missense | G:C>T:A |
| 87 | i7-intron | g.13420G>A | c.782+1G>A | p.? | splice | G:C>A:T |
| 88 | 7-exon | g.13367G>T | c.730G>T | p.G244C | missense | G:C>T:A |
| 89 | i8-intron | g.13900G>A | c.919+1G>A | p.? | splice | G:C>A:T |
| 90 | 10-exon | g.16915C>T | c.1024C>T | p.R342X | nonsense | G:C>A:T at CpG |
| 91 | 8-exon | g.13838A>G | c.858A>G | p.E286E | silent | A:T>G:C |
| 92 | 6-exon | g.12661G>A | c.592G>A | p.E198K | missense | G:C>A:T |
| 93 | 8-exon | g.13861A>G | c.881A>G | p.E294G | missense | A:T>G:C |
| 94 | 7-exon | g.13328A>C | c.691A>C | p.T231P | missense | A:T>C:G |
|  | 8-exon | g.13872del1 | c.892del1 | p.? | frameshift | deletion |
| 95 | 7-exon | g.13371G>A | c.734G>A | p.G245D | missense | G:C>A:T |
| 96 | i9-intron | g.14077T>C | c.993+12T>C | p.? | intronic | A:T>G:C |
| 97 | i8-intron | g.13900G>A | c.919+1G>A | p.? | splice | G:C>A:T |
| 98 | 5-exon | g.12514T>C | c.526T>C | p.C176R | missense | A:T>G:C |
| 99 | 5-exon | g.12443C>T | c.455C>T | p.P152L | missense | G:C>A:T at CpG |
|  | 5-exon | g.12449G>T | c.461G>T | p.G154V | missense | G:C>T:A |
|  | 5-exon | g.12464C>A | c.476C>A | p.A159D | missense | G:C>T:A |
| 100 | 6-exon | g.12715G>A | c.646G>A | p.V216M | missense | G:C>A:T |
| 101 | 7-exon | g.13380G>A | c.743G>A | p.R248Q | missense | G:C>A:T at CpG |
| 102 | 6-exon | g.12655C>T | c.586C>T | p.R196X | nonsense | G:C>A:T at CpG |
| 103 | 7-exon | g.13409G>A | c.772G>A | p.E258K | missense | G:C>A:T |
|  | 8-exon | g.13845C>T | c.865C>T | p.L289F | missense | G:C>A:T |
| 104 | i7-intron | g.13465G>A | c.782+46G>A | p.? | intronic | G:C>A:T |
| 105 | 6-exon | g.12679_12680del2 | c.610_611del2 | p.? | frameshift | deletion |
| 106 | 8-exon | g.13858_13862ins5 | c.878_882ins5 | p.? | frameshift | insertion |
| 107 | 6-exon | g.12682T>C | c.613T>C | p.Y205H | missense | A:T>G:C |

(a): Mutation position in intron/exon (column 2), gene sequence (column 3), cDNA sequence (column 4), codon sequence including one-letter code amino-acid change (column 5) are given, as well as effect of mutation (column 6) and mutation type (column 7). Mutations are annotated as in the IARC TP53 mutation database ([http://www-p53.iarc.fr](http://www-p53.iarc.fr/))

(b): denotes that the effect of the mutation at codon level is not known
