# Supplementary material for: Extremely High Tp53 Mutation Load in Esophageal Squamous Cell Carcinoma in Golestan Province, Iran
Source: PLoS One. 2011 Dec 27;6(12):e29488. doi: 10.1371/journal.pone.0029488 (PMC3246475; doi:10.1371/journal.pone.0029488)
Supplement: Table S3 — Correspondence between TP53 mutation status and Immunohistochemical detection of p53 protein in ESCC cases. (DOC) [file pone.0029488.s003.doc]

**Table S3: Correspondence between *TP53* mutation status and Immunohistochemical detection of p53 protein in ESCC.**

| **p53 Expression**  **No. (%)** | **Mutation Effects** | | | |
| --- | --- | --- | --- | --- |
|  | **Missense** | **Nonsense** | **Splice** | **Frameshift** |
| Negative/Weak | 9 (18.0) | 9 (81.8) | 9(81.8) | 10 (71.4) |
| Moderate/Strong | 41 (82.0) | 2 (18.2) | 2 (18.2) | 4 (28.6) |
| Total | 50 (100) | 11 (100) | 11 (100) | 14 (100) |
| p-value | <0.001 | 0.013 | 0.005 | 0.018 |
